# Gemini Cationic Lipid-Type Nanovectors Suitable for the Transfection of Therapeutic Plasmid DNA Encoding for Pro-Inflammatory Cytokine Interleukin-12

**DOI:** 10.3390/pharmaceutics13050729

**Published:** 2021-05-15

**Authors:** Natalia Sánchez-Arribas, María Martínez-Negro, Clara Aicart-Ramos, Conchita Tros de Ilarduya, Emilio Aicart, Andrés Guerrero-Martínez, Elena Junquera

**Affiliations:** 1 Departamento de Química Física, Facultad de Ciencias Químicas, Universidad Complutense de Madrid, 28040 Madrid, Spain; natsanch@ucm.es (N.S.-A.); mmnegro@ucm.es (M.M.-N.); aicart@ucm.es (E.A.); aguerrero@quim.ucm.es (A.G.-M.); 2 Departamento de Estructura de Macromoléculas, Centro Nacional de Biotecnología, Consejo Superior de Investigaciones Científicas, 28049 Madrid, Spain; caicart@cnb.csic.es; 3 Departamento de Tecnología y Química Farmacéuticas, Facultad de Farmacia y Nutrición, Instituto de Investigación Sanitaria de Navarra (IdiSNA), Universidad de Navarra, 31080 Pamplona, Spain; ctros@unav.es

**Keywords:** non-viral gene delivery, gemini cationic lipid, transfection, protein corona, interleukin-12, pro-inflammatory cytokine

## Abstract

Ample evidence exists on the role of interleukin-12 (IL-12) in the response against many pathogens, as well as on its remarkable antitumor properties. However, the unexpected toxicity and disappointing results in some clinical trials are prompting the design of new strategies and/or vectors for IL-12 delivery. This study was conceived to further endorse the use of gemini cationic lipids (GCLs) in combination with zwitterionic helper lipid DOPE (1,2-dioleoyl-sn-glycero-3-phosphatidyl ethanol amine) as nanovectors for the insertion of plasmid DNA encoding for IL-12 (pCMV-IL12) into cells. Optimal GCL formulations previously reported by us were selected for IL-12-based biophysical experiments. In vitro studies demonstrated efficient pCMV-IL12 transfection by GCLs with comparable or superior cytokine levels than those obtained with commercial control Lipofectamine2000*. Furthermore, the nanovectors did not present significant toxicity, showing high cell viability values. The proteins adsorbed on the nanovector surface were found to be mostly lipoproteins and serum albumin, which are both beneficial to increase the blood circulation time. These outstanding results are accompanied by an initial physicochemical characterization to confirm DNA compaction and protection by the lipid mixture. Although further studies would be necessary, the present GCLs exhibit promising characteristics as candidates for pCMV-IL12 transfection in future in vivo applications.

## 1. Introduction

Interleukin 12 (IL-12) is a promising cytokine in clinical applications due to its biological properties. Considered as a pro-inflammatory cytokine, IL-12 is formed by two covalently linked subunits (a 35-kDa light chain and a 40-kDa heavy chain). It is majorly produced by dendritic cells (DC) and phagocytic cells (monocytes, macrophages and neutrophils) in response to pathogens. Although its mechanism of action is quite complex, scientific studies have contributed greatly to understanding its biological role [1,2,3]. It is clearly one of the main species responsible for the induction and differentiation of cell-mediated immunity [4]. IL-12 increases the activation of cytotoxic T and natural killer (NK) cells and induces the production of other cytokines such as interferon-γ (IFN-γ) [5]. IL-12 has also a critical role in T helper 1 (TH1) response and favors the differentiation and maturation of adaptive immune cells [6]. An efficient and permanent TH1 response seems to be key in autoimmune diseases and/or against infections [7,8]. It has been found that IL-12 expression is rapidly increased by viral infections, such as those caused by hepatitis B virus or human immunodeficiency virus (HIV) [9]. Nowadays, the most seriously ill patients infected by severe acute respiratory syndrome coronavirus 2 (SARS-CoV-2) present abnormal levels of certain cytokines, IL-12 among others [10]. Clinical treatments of COVID-19, the disease caused by SARS-CoV-2 that, at the time of writing this paper, has brought the world to a halt, focus on reducing the levels of pro-inflammatory cytokines. For instance, mesenchymal stem cells (MSCs) have been proposed for the reduction of IL-12 levels [11]. However, COVID-19 seems to be induced not only by an overproduction of inflammatory cytokines, but also by a depletion of antiviral defenses related to the innate immune response [12]. Therefore, to counteract this viral infection, the use of treatments able to either increase the innate immune response or favor differentiation in the adaptive immune response (with a preventive vaccine) is essential [13]. Among the vaccines under development, one based on DNA vaccine technology [14] (which is in the clinical trial phase) combines co-delivery assisted by injection and followed by electroporation of two plasmids DNA (pDNA), one encoding for the SARS-CoV-2 spike (S) protein and the other for IL-12 (pIL-12). At least transiently, mice injected with both pDNAs showed an increase of the total neutralizing antibodies detected against SARS-CoV-2 relative to those injected with only pDNA encoding for the SARS-CoV-2 spike (S) protein [15].

On the other hand, IL-12 is also interesting for its antitumor activity. This cytokine activates different pathways in resistance to tumor growth where innate and adaptive immune cells and others pro-inflammatory cytokines, such as IFN-γ, are involved [16]. The IFN-γ production induced by IL-12 exerts toxic effects on tumor cells and activates potent anti-angiogenic mechanisms [17]. Therefore, scientists have been interested in the use of IL-12 as a candidate for tumor immunotherapy [18]. Many IL-12 delivery strategies have been successfully carried out in different animal models [18,19]. However, the encouraging results found in in vivo models have not been confirmed in clinical trials. In fact, in initial studies, the observed toxicity levels were completely unexpected after systemic dosage with recombinant IL-12 [20]. One of the principal approaches to deliver IL-12 is based on gene therapy strategies, where a polynucleotide encoding for IL-12 is introduced into the tumorous cell by viral (e.g., adenovirus [21], retrovirus [22] and alphavirus [23]) or non-viral vectors (e.g., polymers [24], cationic lipids, CLs [25] and lipopolymers) [26]. Alternatively, pDNA encoding for IL-12 has also been intratumorally or intramuscularly injected followed by electroporation [27]. Promising approaches have combined IL-12 gene therapies with other therapeutic modalities. For instance, all-trans-retinoic acid has been applied with cationic liposomes/pIL-12, resulting effective for the inhibition of metastatic lung tumors in mice [28]. Nowadays, improved techniques and novel vectors for IL-12 delivery that allow IL-12 production to be restricted and sustained at the tumor site are required [19]. IL-12-based gene therapies seem to be a safe option for local and prolonged production of IL-12, reducing the adverse effects provoked by the stimulation of IFN-γ. However, the immunosuppressive tumor microenvironment in humans limits the clinical efficiency of IL-12. Therefore, during the design of nanocarriers for IL-12, it is necessary to consider the different biological environments where IL-12 will be delivered. In addition, the possible interactions with biomolecules present in those media must be taken into account. For instance, it is well-known that opsonin proteins trigger an immune response and condition the circulation time of vectors [29,30]. Indeed, nanomedicine focuses on the protein coating around nanomaterials, named protein corona (PC), which provides the nanovector with a new biological identity and determines its target [31,32,33,34,35,36].

**Scheme 1:**
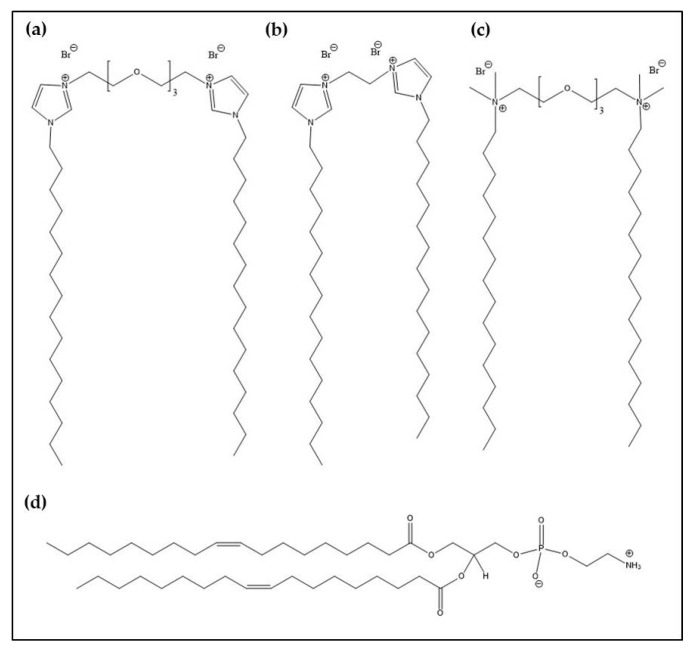
Molecular structure of (**a**) GCL1, (**b**) GCL2 and (**c**) GCL3. The molecular structure of the zwitterionic lipid DOPE is shown in (**d**).

With all this in mind, the present work was envisioned to design alternative non-viral vectors for pIL-12 and evaluate the influence of the adhered proteins in a biological environment. To achieve this aim, a series of gemini cationic lipids (GCLs) previously developed in our group as very efficient and safe non-viral vectors of a plasmid encoding for the green fluorescent protein (pEGFP-C3) [37,38,39] are here proposed to transfect a reporter gene (encoding for luciferase) and a therapeutic gene (encoding for pro-inflammatory cytokine IL-12) to living cells. GCLs (twin lipid blocks each formed by a long hydrocarbon chain linked to a positively charged head and further joined by a spacer) are a well-known group of cationic nanovectors that have widely demonstrated their potential in gene transfection [40,41,42]. Their low toxicity and broad structural versatility (due to possible changes in the polar heads and/or the hydrocarbon chains and/or the spacer) are their principal advantages [43,44,45]. In addition, their fusogenic properties with cell membranes can be enhanced by combination with a coadyuvant zwitterionic lipid, i.e., 1, 2-dioleoyl-sn-glycero-3-phosphatidyl ethanol amine, DOPE. In particular, the GCLs used in this work are (i) 1,2-bis(hexadecyl dimethyl imidazolium) trioxyethylene [37] (hereinafter denoted as GCL1), (ii) 1,2-bis(hexadecyl imidazolium) dialkane [38] (GCL2) and (iii) 1,2-bis(hexadecyl dimethyl ammonium) trioxyethylene [39] (GCL3) (see Scheme 1 for structural details). The lipid mixtures were completed in all cases with the addition of zwitterionic lipid DOPE at the optimal ratio for each lipoplex, i.e., those at which the resulting lipoplexes show low cytotoxicity and the best transfection efficiency, even superior to that obtained with the universal control Lipofectamine2000* [37,38,39]. The lipoplexes formed with the lipid mixtures and the pDNAs herein used were initially physicochemically characterized by ζ-potential, agarose gel electrophoresis and cryo-transmission electron microscopy (cryo-TEM) analyses. These experiments were carried out (i) to confirm the DNA complexation, (ii) to evaluate the DNA compaction and protection ability of the lipid mixture and (iii) to determine the morphology and structure of the lipoplexes. Secondly, nano-liquid chromatography/mass spectrometry (nanoLC-MS/MS) was used to determine the nature of the PC formed around the lipoplexes incubated in human serum (HS). This provides valuable information on the preferences of the lipoplex surface toward human proteins. Since the lipoplexes must be cell-friendly, alamarBlue assays were performed in selected cell lines to evaluate the cell viability. The transfection efficiency of the vectors was firstly determined with luminometry, using pDNA encoding for a luciferase protein. Finally, enzyme-linked immunosorbent assays (ELISA) revealed the transfection capacity of pDNA encoding for IL-12 achieved by the chosen vectors. Therefore, this study highlights the capacity of GCL-type nanovectors to transfect this promising cytokine, in an attempt to contribute to the advancement of delivery techniques for IL-12 that could in turn improve the results obtained so far in clinical trials and/or in in vivo applications.

## 2. Materials and Methods

### 2.1. Materials

The synthesis of GCLs has been previously reported [37,38,39]. Zwitterionic lipid DOPE was supplied by Avanti Polar Lipids, Inc. (Alabaster, AL, USA). The transfection efficiency was determined using two plasmids: (i) pCMV-Luc VR1216 plasmid DNA (6934 bp) encoding for luciferase (pCMV-Luc), which was supplied by Clontech Laboratories (Palo Alto, CA, USA) and amplified in *E. coli*, using a Qiagen Plasmid Giga Kit (Qiagen GMBH, Hilden, Germany); and (ii) therapeutic pCMV-interleukin-12 (5500 bp) encoding for interleukin-12 (pCMV-IL12), which was kindly donated by Dr. Chen Qian (University of Navarra, Pamplona, Spain). Dulbecco’s modified Eagle’s medium (DMEM) high glucose + Gluta-MAX was supplied by Gibco-ThermoFisher (Waltham, MA, USA). Human serum (HS) was used as received from Sigma-Aldrich (St. Louis, MO, USA). The commercial control Lipofectamine2000* Transfection Reagent, abbreviated as Lipo2000*, was purchased from Invitrogen-ThermoFisher (Waltham, MA, USA).

### 2.2. Preparation of Lipoplexes

In order to obtain the desired GCL molar composition in the mixed lipids (α), corresponding amounts of each GCL and zwitterionic lipid DOPE were dissolved in chloroform and, finally, dry lipid films were formed by evaporation of the solvent under high vacuum. These dry films were hydrated and homogenized in *N*-(2-hydroxyethyl)piperazine-*N*′-ethanesulfonic acid (HEPES)-buffered medium (40 mM, pH = 7.4) by alternating vortex/sonication cycles. The obtained multilamellar lipid mixtures were passed through a sequential extrusion procedure detailed elsewhere to obtain unilamellar lipid mixtures with ~100 nm of size [46]. Different amounts of these solutions were combined with specific amounts of pDNA to form lipoplexes at specific effective charge ratios (ρ_eff_). Depending on the experimental technique, the pDNA stock solution concentration varied as follows: 0.1 mg/mL for ζ-potential and agarose gel electrophoresis, 0.2 mg/mL for cryo-TEM and protein corona studies and 0.1 µg/mL for biological experiments.

### 2.3. Physicochemical Characterization Methods

The ζ-potential of the GCL/DOPE-pDNA lipoplexes was determined at 25 °C through the electrophoretic mobility measured by the phase analysis light scattering technique (Zeta PALS, Brookhaven Instruments Corp., Holtsville, NY, USA) following a procedure fully described elsewhere [47,48]. The experimental conditions were 1.33 of dispersant refractive index of water, 0.9 cP of viscosity and 78.5 of dispersant dielectric constant. The particle size of lipoplexes was determined from dynamic light scattering (DLS) measurements carried out on a particle analyzer Zeta Nano Series (Malvern Instruments, Barcelona, Spain). Both properties, ζ-potential and particle size, were calculated as the average of 50 and 30 independent measurements, respectively, as a function of the mass ratio between the lipid mixture and pDNA (m_L_/m_pDNA_) or effective charge ratio (ρ_eff_), given by Equations (1) and (2):

(1) $$m_{L}{/m}_{\mathrm{pDNA}}=\;{(m}_{\mathrm{GCL}}{+m}_{L^{0}}{)/m}_{\mathrm{pDNA}}$$

where m_L_, m_GCL_, m_L^0^_ and m_pDNA_ are the masses of the total mixed lipid, GCL, zwitterionic lipid (DOPE) and pDNA, respectively.

(2) $$\rho_{\mathrm{eff}}=\frac{n^{+}}{n^{-}}\;=\frac{q_{\mathrm{eff},\mathrm{GCL}}^{+}{(m}_{\mathrm{GCL}}{/M}_{\mathrm{GCL}})}{q_{\mathrm{eff},\mathrm{pDNA}}^{-}{(m}_{\mathrm{pDNA}}{/M}_{\mathrm{pDNA}})}$$

where n^+^, n^−^, $q_{\mathrm{eff},\mathrm{GCL}}^{+}$, $q_{\mathrm{eff},\mathrm{pDNA}}^{-}$, M_GCL_ and M_pDNA_ are the number of moles of positive (GCL) and negative (pDNA) charges, effective charges of GCL and pDNA per bp, and the molecular weight of GCL and pDNA per bp, respectively.

Agarose gel electrophoresis was used to determine the capacity of the lipid mixtures to compact and protect pDNA. To evaluate DNA compaction, lipoplexes were formed and loaded in agarose gel (0.8% (*w*/*v*) in 1× TAE with 0.7 µL of GelRed). For the evaluation of DNA protection, lipoplexes were previously incubated for 2 h at 37 °C in DNase I medium (1 U/µg DNA). To inactivate the DNase action, 4 µL of 0.25 M EDTA was added to the samples for 15 min. Then, to disrupt the lipoplexes, 15 µL of 25% sodium dodecyl sulfate (SDS) was included. After 5 min, the samples were loaded on the agarose gel (1% (*w*/*v*) in 1× TBE with 3 µL EtBr). In both cases, electrophoresis was run at 70 mV for 1 h and visualized using an XR instrument (Bio-Rad, Hercules, CA, USA) and GelDoc software (Bio-Rad), excited at 302–312 nm and recorded at 600 nm (for GelRed probe), or excited at 482 nm and recorded at 616 nm (for EtBr).

Cryo-TEM experiments were carried out following the standard protocol [49,50,51]. Briefly, an ionic discharge was applied for 1 min (Agar Scientific Ltd., Stansted, UK) on perforated Holey Carbon on a 400-mesh copper grid, where a drop of lipoplexes was previously deposited. The grid was mounted on a piston (Leica EM GP) and immediately vitrified by rapid immersion in liquid ethane at −180 °C on a Gatan 626 cryo-transfer system (Gatan Inc., Pleasanton, CA, USA). Micrographs were recorded with a Gatan 794 Ultrascan digital camera and analyzed with the digital Micrograph software using a JEOL JEM 2011 microscope at 200 kV under low-dose conditions and different degrees of defocus (500–700 nm).

### 2.4. Protein Corona Studies

The procedure for the analysis of the protein corona surrounding the surface of lipoplexes using nanoLC-MS/MS was fully detailed in a previous work [52]. Briefly, GCL/DOPE-pDNA lipoplexes were incubated in HS medium at 37 °C for 1 h. After centrifugation (14,000 rpm, 5 min), washing and resuspension, the samples were subjected to dithiothreitol reduction and iodacetamide alkylation before a trypsin digestion treatment (at 37 °C, overnight). The peptides thus obtained were acidified and subsequently eluted, concentrated and desalted by C18 reverse phase chromatography using silica SPE C18 commercial columns (OMIX C18), following the instructions of the manufacturer for the packaging and conditioning of the column. The samples were dried by vacuum centrifugation (SpeedVac) and reconstructed in formic acid for their analysis in a nano Easy-nLC 1000 system, fitted to a Q-Exactive HF high resolution mass spectrometer through a Nano-Easy spray source. Peptides were separated in an Acclaim PepMap 100 Trapping pre-column and eluted onto a NTCC C18 resin analytical column at a constant flow rate (250 nL/min). Then, the peptides were electrospray ionized using an ion spray voltage of 1.8 kV at 250 °C. The MS/MS spectra were acquired in positive mode and analyzed with the search engine Sequest with the Proteome Discoverer 2.2 software. The percolator algorithm was used to estimate the false discovery rate (FDR) <1% and only the identification of proteins that had been correlated to at least two unique peptides based on mass spectra were considered. For the quantitative analysis of proteins, the mean value of peptide to spectrum matches (PSMs) was normalized to the molecular weight (MW) of the protein in kDa and expressed as the relative percentage of protein.

### 2.5. In Vitro Studies

HeLa (human cervix adenocarcinoma) and COS-7 (African green monkey kidney fibroblast like) cells were purchased from American Type Collection (Rockville, MD, USA) and kept under standard culture conditions (37 °C, 5% CO_2_) in DMEM-high glucose + Gluta-MAX supplemented with 10% (*v*/*v*) heat-inactivated fetal bovine serum (FBS), 100 U/mL penicillin and 100 μg/mL streptomycin. For the in vitro experiments, cells were seeded in 48-well plates (1 mL, 10^5^ cells/well). After 24 h, lipoplexes formed at 1 µg DNA/well were incubated with the cells for 4 h. Next, the culture medium was renewed and maintained until the cell viability and transfection quantification experiments were performed. The same protocol and conditions were used for the positive control, Lipo2000* (1.5 µL/µg DNA).

The cytotoxicity of the GCL/DOPE-pDNA lipoplexes was determined by a modified alamarBlue assay. Briefly, after 48 h of lipoplex incubation, cells were incubated during 2 h with 1 mL of 10% (*v*/*v*) alamarBlue dye in DMEM medium. Then, 200 µL of the supernatant was collected from each well and transferred into a 96-well plate suitable for a Power Wave XS plate reader, where the absorbance at 570 nm and 600 nm was measured. The % of cell viability for the samples was determined by using the following Equation:

(3) $$100\times \left[{\left(A_{570}-A_{600}\right)}_{\mathrm{treated}\;\mathrm{cells}}/{\left(A_{570}-A_{600}\right)}_{\mathrm{untreated}\;\mathrm{cells}}\right]$$

In this work, the transfection efficiency of the lipoplexes was quantified by Luminometry (for pDNA encoding for luciferase) and by Enzyme-Linked ImmunoSorbent Assay, ELISA (for pDNA encoding for IL-12).

#### 2.5.1. Luminometry

After 48 h of lipoplex incubation, the cells were washed with phosphate-buffered saline (PBS) and lysed with 100 µL of the lysis buffer (Promega, Madison, WI, USA) for 10 min, at room temperature, followed by two consecutive freeze–thaw cycles. The lysed cells were collected and centrifuged (1200 rpm, 5 min). From the supernatant, 20 µL was taken for the luciferase assay with the Luciferase Assay Reagent (Promega), using a Sirius-2 luminometer (Berthold Detection System, Innogenetics, Diagnóstica y Terapéutica, Barcelona, Spain), and 10 µL was for the protein assay by Bio-Rad DC Protein Assay (Bio-Rad Laboratories, Hercules, CA, USA). The absorbance was recorded at 690 nm with a Power Wave XS plate spectrophotometer and a KC junior data processor, BioTekS, using bovine serum albumin (BSA) as standard. Therefore, the transfection efficiency was evaluated in terms of the RLU/mg protein, which was converted to ng luciferase/mg protein, using a standard curve.

#### 2.5.2. Enzyme-Linked Immunosorbent Assay

The IL-12 cytokine levels in cells were determined by using the Mouse IL-12 (p70) ELISA Set kit, BD Biosciences Pharmingen (San Diego, CA, USA), following the manufacturer’s instructions. Briefly, anti-mouse IL-12 (p70) monoclonal antibody was fixed in the 96-well plate, overnight, at 4 °C. The assay diluent was added and incubated at room temperature, for 1 h, to block the plate. Next, 100 µL of the cell medium supernatant of the samples and the standard solutions (recombinant mouse IL-12 (p70)) were incubated for 2 h, at room temperature. Then, 100 µL of the working detector solution was added and incubated for 1 h. This working solution contained the second biotinylated anti-mouse IL-12 (p70) monoclonal antibody as the detection antibody and the streptavidin-horseradish peroxidase conjugate (SAv-HRP) as the enzyme reagent. Manual plate washings were performed between each step. Finally, 100 µL of the substrate solution was added and oxidized by the enzyme reagent by dark incubation for 30 min. The reaction was stopped to measure the absorbance of samples at 450 and 570 nm, using a Power Wave XS plate spectrophotometer and a KC junior data processor, BioTekS. The IL-12 concentration of samples, expressed in ng IL-12/mL, was obtained through the standard curve run in the assay.

## 3. Results and Discussion

Basic science studies have proven to be extremely helpful to approach global health problems such as cancer, infections and, in particular, the COVID-19 pandemic. The fast but reliable and robust response against SARS-CoV-2 by the scientific community is one example of how crucial is not only to develop safe health platforms with promising effectiveness but also to advance in the understanding of the main principles and mechanisms involved in them. In the case of IL-12, many studies have demonstrated its efficacy in animal models as an anticancer agent or against different infections, as summarized in the Introduction section. However, the complex and different biological responses observed in patients treated with diverse strategies employing this cytokine highlights the need for further studies to expand our understanding. In all cases, the problem of how to reach the target cells by using a cell-friendly strategy with the highest efficiency remains a bottleneck. Based on our expertise in the field of lipofection, we have faced this work by selecting, among the many cationic nanovectors characterized by our group (and in some cases even designed and synthesized), three of the best candidates for the insertion of pCMV-IL12 in HeLa and COS-7 cell lines, with a previous characterization study with a reporter gene pCMV-Luc. Thus, GCLs denoted as GCL1, GCL2 and GCL3 (see Scheme 1), mixed with DOPE, were selected at their optimal formulations, i.e., α = 0.2 for GCLs with an imidazole head group (GCL1 and GCL2), and α = 0.7 for the GCL with a quaternary ammonium head group (GCL3).

### 3.1. Physicochemical Characterization

**Figure 1:**
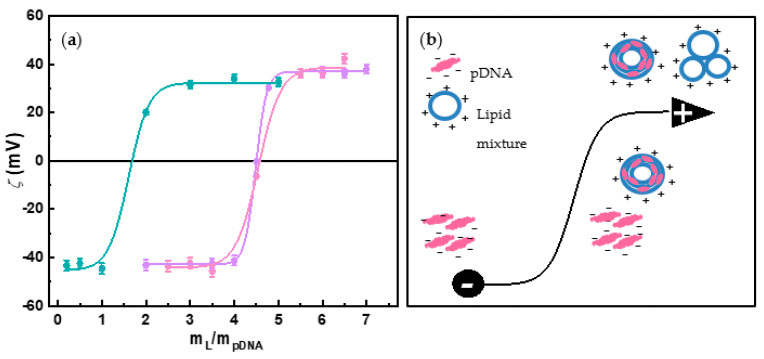
(**a**) Plot of the ζ-potential as a function of the $m_{L}{/m}_{\mathrm{pDNA}}$ mass ratio for the optimum formulations of the GCL/DOPE-pCMV-Luc lipoplexes: GCL1/DOPE-pCMV-Luc (purple line), GCL2/DOPE-pCMV-Luc (pink line) and GCL3/DOPE-pCMV-Luc (blue line). (**b**) Schematic drawing of the charge inversion process.

One of the conditions that seems to be essential to obtain nanovectors with promising effectiveness and efficient transfection ability is that they have to be positively charged in order to complex and compact anionic polynucleotides. This favors their absorption by the cell membrane, usually following an endocytic pathway, and aids their crossing of this barrier. During lipoplex formation, this condition is achieved once electroneutrality is overpassed and the nucleic acids are totally compacted by the lipid mixture. ζ-potential measurements vs. the $m_{L}{/m}_{\mathrm{pDNA}}$ ratio provide this information from the charge inversion observed in the sigmoidal curves of Figure 1a (see Figure 1b for a schematic drawing of this charge inversion process). Table S1 includes the electroneutrality values obtained for the three different GCL/DOPE mixtures, as well as the effective charges of both pDNA ($q^{-}{}_{\mathrm{eff},\mathrm{pDNA}}/\mathrm{bp}$) and GCLs ($q^{+}{}_{\mathrm{eff},\mathrm{GCL}}$) [37,38,39]. These effective charges have been determined using a protocol fully described elsewhere [38]. Note that no significant differences exist in the $q^{-}{}_{\mathrm{eff},\mathrm{pDNA}}/\mathrm{bp}$ values obtained for the three lipoplexes (−1.4 ± 0.1/bp on average), being in all cases around 30% less negative than the nominal ones (−2.0/bp). This difference between effective and nominal charges for pDNA reveals that a significant fraction of Na^+^ counterions remain associated with the phosphate groups of pDNA once the lipoplex adopts a supercoiled conformation at physiological pH. Accordingly, those other Na^+^ counterions that are expelled to the bulk contribute to a clear entropy gain, being this entropy factor an important driving force toward lipoplex formation, together with the significant electrostatic interaction between the cationic lipidic nanovector and anionic pDNA. This feature is advantageous for our purposes since less amount of GCL (around ~30% than that expected if $q_{\mathrm{pDNA}}^{-}$ = −2) is required to prepare positively charged lipoplexes with pDNA, which may result in lower lipoplex toxicity. In what respects to the GCL effective charge, the previously reported values were around 10% less positive than the nominal ones (+2) [37,38,39]. Both $q^{-}{}_{\mathrm{eff},\mathrm{pDNA}}$ and $q^{+}{}_{\mathrm{eff},\mathrm{GCL}}$ were used to prepare lipoplexes at the desired effective charge ratios ρ_eff_ (Equation (2)) in all the experiments herein reported.

**Figure 2:**
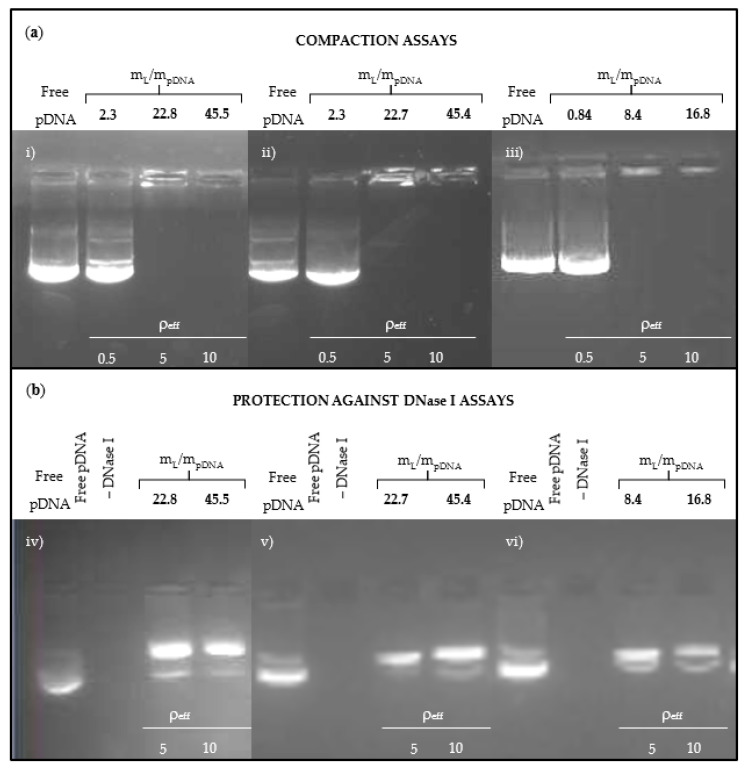
Agarose gel electrophoresis experiments of GCL/DOPE-pCMV-Luc lipoplexes at selected optimal conditions of $m_{L}{/m}_{\mathrm{pDNA}}$ mass ratios and/or ρ_eff_. Panel (**a**) shows the compaction assays, where free pDNA (lane 1) was used as the control. Panel (**b**) presents the protection assays of pDNA against degradation by DNase I, where free pDNA (lane 1) and free pDNA incubated with DNase I (lane 2) were used as controls. For both agarose electrophoresis experiments: (i) and (iv) correspond to the GCL1/DOPE-pCMV-Luc system, (ii) and (v) correspond to the GCL2/DOPE-pCMV-Luc system, and (iii) and (vi) correspond to the GCL3/DOPE-pCMV-Luc system.

The compaction of pDNA can also be evaluated by agarose gel electrophoresis. In these experiments, the GelRed probe dispersed in the agarose gel is intercalated within the hydrophobic environment of the double-stranded helixes of DNA, increasing the quantum emission yield of the probe. These fluorescent bands are not observed if the DNA is compacted by the lipid mixture. Figure 2a shows the agarose gel electrophoresis of the three GCL/DOPE-pDNA lipoplexes. In all cases, at low $m_{L}{/m}_{\mathrm{pDNA}}$ values (lane 2, ρ_eff_ = 0.5), the lipid mixtures are not able to compact pDNA and the fluorescent bands are visualized, as in the control lane with free DNA (lane 1). However, at higher $m_{L}{/m}_{\mathrm{pDNA}}$ values (lanes 3 and 4 for ρ_eff_ = 5 and 10, respectively), the absence of the bands confirms the full compaction of pDNA by the mixed lipid-based nanovector. This is in agreement with the ζ-potential results previously discussed. Furthermore, agarose gel electrophoresis was not only used in this work to determine the ability of the mixed lipid-based nanovectors to complex and compact pDNA, but also to evaluate the protection they provide to the nucleic acid against degradation by the DNase I enzyme normally present in biological fluids (Figure 2b). In this experiment, two controls were used: free pDNA in the absence (lane 1) and presence of DNase I (lane 2). Due to the degradation of pDNA, the fluorescent bands seen in lane 1 are not observed in lane 2. The same incubation experiments in DNase I medium were also performed for the lipoplexes formed at ρ_eff_ = 5 and 10 (lanes 3 and 4, respectively). After treatment with SDS, the mixed lipid nanoaggregates are disrupted and pDNA is recovered intact, as can be deduced by the presence of the pDNA characteristic bands, thus confirming the protective action of the lipid-type nanovectors against DNase I. After a quick glance to lanes 3 and 4 in Figure 2, it is interesting to notice how the formulations at which pDNA is efficiently compacted by the lipid-based nanovector (i.e., no fluorescent bands are seen along the lanes in the upper panels) are also those in which the nucleic acid finds a protecting environment against the degrading external medium (i.e., the fluorescent bands are seen after disruption of the lipidic nanoaggregate in the lower panels).

**Figure 3:**
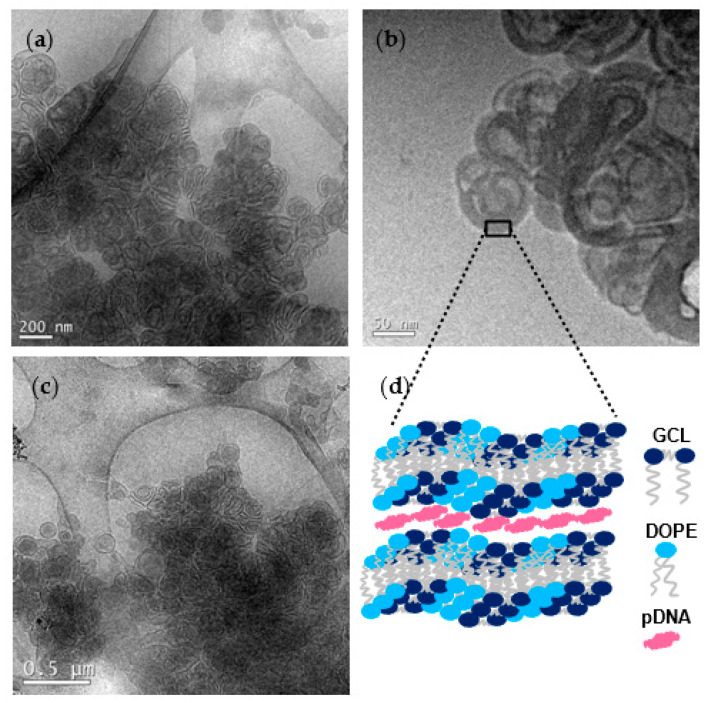
Selection of cryo-TEM micrographs of GCL1/DOPE-pCMV-Luc lipoplexes formed at ρ_eff_ = 5. The scale bars of the micrographs are 200 nm (**a**), 50 nm (**b**) and 500 nm (**c**). Panel (**d**) shows a scheme of the L_α_ multilamellar phase observed in the micrographs.

The lipoplexes were evaluated also from a structural point of view before in vitro studies to confirm their suitability in terms of structure and size to cross the cell membrane, which is one of the critical steps in the transfection process. The morphology and structure of the GCL/DOPE-pDNA lipoplexes were visualized by cryo-TEM analysis. The micrographs in Figure 3 and Figure S1 show the multilamellar-type arrangement of GCL/DOPE-pDNA lipoplexes, as previously found in GCL/DOPE-pEGFP-C3 lipoplexes [37,38,39] and similar nanovectors published in the literature [53,54,55]. These L_α_ multilamellar lyotropic liquid crystal phases are characterized by an alternating configuration of lipidic bilayers of positively charged GCL/DOPE mixed lipids and aqueous monolayers, where the anionic pDNA helixes are located in a sandwich-type fashion (see scheme on Figure 3d). On the other hand, the particle size (hydrodynamic diameter, D_h_) and polydispersity index (PDI) of the lipoplexes at two conditions (ρ_eff_ = 5 and 10) were determined by DLS (see Table 1). Sizes of ~200 nm and polydispersities below 0.4, such as those reported in Table 1, are typically considered acceptable for efficient crossing through the cell membrane and, subsequently, for the transfection process [56,57].

### 3.2. Protein Corona Characterization

**Figure 4:**
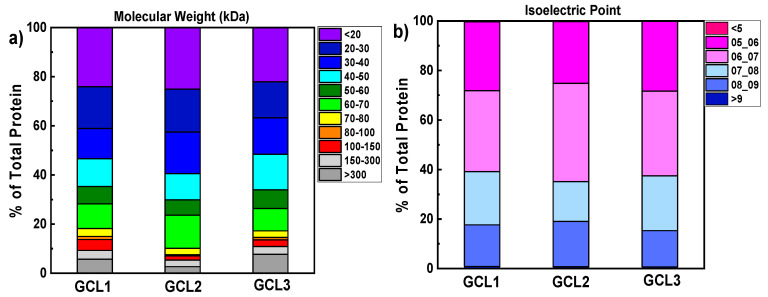

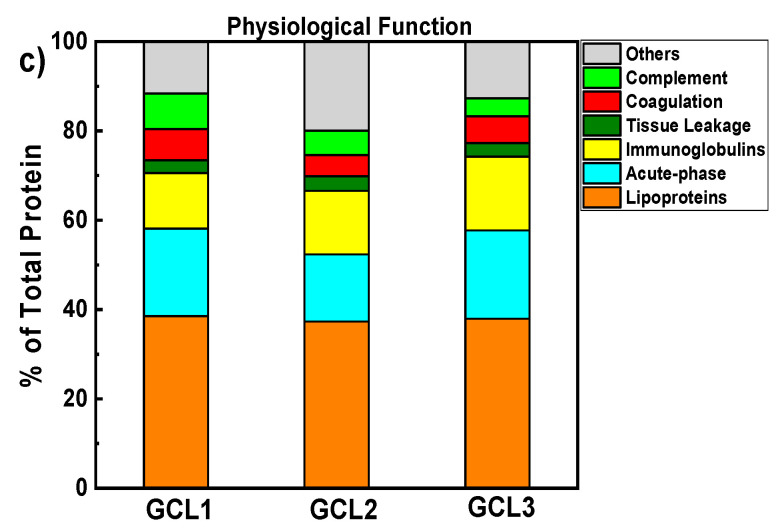
Relative percentage of the proteins found in the protein corona (PC) of the GCL/DOPE-pCMV-Luc lipoplexes at ρ_eff_ = 5, classified by (**a**) molecular weight, MW in kDa; (**b**) isoelectric point, pI; and (**c**) physiological function.

The fate of a nanovector depends largely on the type and amount of proteins that adhere to its surface when it comes into contact with a biological fluid; that is, when it enters the bloodstream. In fact, what cells really see is the new entity formed by the lipoplex surrounded by the adsorbed PC, and it is this PC the cells interact with rather than the lipoplex itself. Accordingly, the evaluation of the protein corona (in terms of its nature and composition) is crucial to understand the in vitro behavior of these non-viral vectors and even to estimate their potential success in vivo. The adsorption of proteins (PC formation) has special interest since one of the main barriers for nanovectors to reach the target cells is the opsonization process. Certain proteins can inhibit this process and increase the bloodstream time circulation, while those in charge of cellular recognition can even favor the passage through the cellular membrane. For that reason, in this work, lipoplexes formed by either GCL1, GCL2 or GCL3, combined with DOPE and pCMV-Luc at ρ_eff_ = 5 were incubated in HS and analyzed by nanoLC-MS/MS to determine the proteomic profile of their PC. A large number of proteins were found in all cases: 161 for GCL1/DOPE-pCMV-Luc, 143 for GCL2/DOPE-pCMV-Luc and 144 for GCL3/DOPE-pCMV-Luc, of which 127 were common to all of them. The presence of such a significant number of proteins in the PC has also been reported for PEGylated liposomes [58]. The relative percentages of the different proteins were calculated and subsequently classified according to their molecular weight (MW), isoelectric point (pI) and physiological function, as reported in Figure 4a–c, respectively (see also Table S2). The results show only 3–8% of proteins with a MW higher than 300 kDa, being proteins with MW < 70 kDa those predominantly adsorbed on the nanocarrier surface (82–90%), in agreement with those observed in a previously reported C_3_(C_16_His)_2_/MOG-siRNA nanovector with histidine-based polar heads [59]. Moreover, around 61–65% of adsorbed proteins present pI < 7, i.e., they bear a net negative charge at physiological pH, indicating that, as found in previous studies [52,59], strong electrostatic interaction between these proteins and the positively charged nanovectors is probably one of the main non-covalent intermolecular forces driving the PC formation process. In what respect to physiological function, lipoproteins were preferentially adsorbed on the surface of the three lipoplexes (37–39%), followed by acute-phase proteins (15–20%) and immunoglobulins (Igs) (12–17%). Igs and acute-phase proteins are considered responsible for the immune system response, recognizing pathogens and binding them to trigger the appropriate immune process. In contrast, a lipoprotein coverage is usually associated with improved bloodstream circulation times. Lipoproteins are related to intercellular transport of insoluble lipids, particularly cellular processes involved in cholesterol metabolism. On the other hand, tissue leakage, coagulation and complement proteins were those found in minor proportion (4–9%, 4–6% and 4–7%, respectively). Complement and coagulation proteins also participate in the innate immune response. By binding pathogens directly or indirectly (through antibodies), complement proteins induce an inflammatory response and the recognition by phagocytes, thus favoring the elimination of nanocarriers from systemic circulation. Although they are mainly in charge of maintaining hemostasis (i.e., blocking a bleeding process), coagulation proteins can also physically trap microbes or stimulate the opsonization process around pathogens. On the other hand, cellular death or damage provokes the release of tissue leakage proteins to the blood; therefore, they are found in lower concentration in blood than classical proteins such as Igs or coagulations proteins. The proteins identified for each physiological function are shown in detail in Figure S2 (major fraction) and Figure S3 (minor fraction). The slight differences found in the PC of the three lipoplexes are necessarily attributed to the diverse nature of the gemini cationic lipid (head and spacer group). These structural differences are expected to be rather subtle from the perspective of the adhering proteins. Moreover, the content in DOPE does not seem to drastically influence the PC composition, notwithstanding that zwitterionic species usually avoid protein adsorption [60].

The top 25 most abundant proteins found on surface of the three lipoplexes, also classified by MW, pI and physiological function (Supplementary Materials Figure S4), are consistent with the total protein results. These 25 most abundant proteins (~70–80% of the total proteins) are collected in Table 2. The color scale shows the decreasing percentage from intense red to yellow and finally gray. The most abundant proteins on the surface of GCL1/DOPE-pCMV-Luc lipoplex were trypsin (6%), apolipoproteins (B-100, A-II, A-I and C-I, with ~5% each one) and serum albumin (~5%). Serum albumin was the main protein (~11%) for the GCL2/DOPE-pCMV-Luc lipoplex, followed by apolipoproteins A-I and A-II, trypsin, and protein AMBP (around 6–7% each). Finally, apolipoproteins (B-100, A-I and A-II), serum albumin and trypsin were the most abundant proteins found in the case of the GCL3/DOPE-pCMV-Luc lipoplex (around 6–8% each). Generally speaking, the three lipoplexes share the same most abundant proteins, apolipoproteins A-I (APO A-I) and A-II (APO A-II), serum albumin and trypsin, albeit with slight differences in their relative percentages.

As it was mentioned previously, lipoproteins such as apolipoproteins seem to increase the blood circulation time of nanovectors and favor the interactions with low density lipoprotein (LDL) receptors, which allows crossing the brain barrier [61]. Serum albumin is the most abundant protein in blood and it contributes to transport certain molecules due to its ability to avoid recognition and subsequent uptake by macrophages. In addition, it seems to promote the accumulation of nanocarriers in tumors [62], which can be interesting for the local delivery of IL-12 in the context of anticancer applications. In many solid tumors, the vitronectin receptor is overexpressed, so the presence of vitronectin among the 25 most abundant proteins for the GCL1/DOPE-pCMV-Luc and GCL2/DOPE-pCMV-Luc lipoplexes may result in improved uptake by cancerous cells [63]. On the other hand, certain proteins such as complement C3 protein, belonging to the opsonins group, was not found among the 25 most abundant proteins for the GCL2/DOPE-pCMV-Luc lipoplex or found at low relative percentages for GCL1/DOPE-pCMV-Luc and GCL3/DOPE-pCMV-Luc (~1%), this feature being in principle positive in terms of potentially longer systemic circulation of the nanovectors.

Finally, although the 25 most abundant proteins in the PC of each lipoplex do not show significant differences for us to predict better or worse results in cell cultures, the high albumin content around the GCL2/DOPE-pCMV-Luc lipoplex may provide some potential advantage over the other two. On the other hand, the PC around GCL3/DOPE-pCMV-Luc lipoplex showed a higher content of Igs, which may condition its ability to reach the target in a biological environment. Other lipoplexes constituted by small interfering RNAs and lipid mixtures with GCL or CL (which included histidine or arginine residues on the polar heads) also showed APO A-I and A-II and serum albumin as their main PC fractions [52,59]. This indicates that, even when using different lipids, similar human proteins are adsorbed on the surface of the lipoplexes. This evidence, which we have been observing in our latest studies with lipoplexes, can be of great help when developing strategies for cell transfection. In summary, the high content of lipoproteins and serum albumin led us to expect good results in intracellular trafficking in biological fluids. Nevertheless, these results were not significant enough to discard any of the lipoplexes before the in vitro studies.

### 3.3. In Vitro Studies

**Figure 5:**
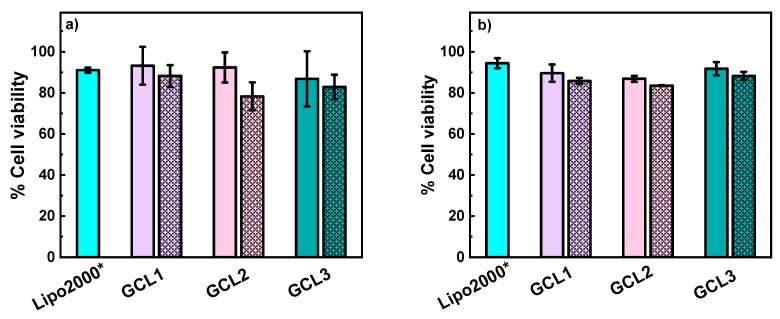
Percentage of cell viability of COS-7 (**a**) and HeLa (**b**) cell lines in the presence of the optimized formulations of the GCL/DOPE-pCMV-Luc lipoplexes formed at ρ_eff_ = 5 (unstriped bars) and 10 (striped bars). The lipoplexes were incubated with cells for 48 h. The cell viability (%) was normalized to that obtained for untreated cells. The blue bar corresponds to commercial Lipo2000*, used here as positive control. Data represent the mean of three wells and errors are within ±5%.

Cellular transfection is always a complex process and typically one of the limitations in the use of synthetic non-viral vectors vs. viral vectors. However, clinical trials on IL-12 administration have used different non-viral vector based-strategies to improve the antitumor ability of this cytokine, thus avoiding the risks associated with viral vectors. Control over the toxicity together with the necessity of minimizing possible adverse effects provoked by the vectors are always a priority in any treatment. Unexpected toxicity is usually the main reason preventing the development of certain tests, as occurred at the beginning of IL-12 therapies [20]. Although still far from clinical trial phases, in vitro experiments in cell lines, such as those reported in this study, are the previous steps to discard nanovectors that may exert severe toxicity. In this context, the possible cytotoxicity of the GCL/DOPE-pCMV-Luc lipoplexes was evaluated. After 48 h of incubation in COS-7 and tumor HeLa lines, the metabolic activity of cells was measured by means of alamarBlue assays and expressed in terms of % cell viability, as shown in Figure 5a,b for COS-7 and HeLa cells, respectively. The reagent Lipo2000*, incubated under the same conditions, was here used as universal positive control. As shown in the figure, the % of cell viabilities were above 80% in both cell lines for the three lipoplexes studied at two selected conditions, ρ_eff_ = 5 (unstriped bars) and ρ_eff_ = 10 (striped bars).

No significant differences were observed, i.e., neither the dissimilar polar head (imidazole vs. quaternary ammonium) nor the different DOPE content seemed to influence the cellular proliferation. However, a very slight decrease in the cell viability values was observed for each GCL/DOPE-pCMV-Luc lipoplex at ρ_eff_ = 10 with respect to ρ_eff_ = 5, as also seen in other lipoplexes in the literature [52,53]. Either way, the results corroborate the excellent biocompatibility of these three GCL-based lipoplexes, also previously observed with the plasmid encoding for pEGFP-C3 in several cell lines [37,38,39]. With the results obtained in this preliminary study in hand, all three GCL/DOPE-pCMV-Luc lipoplexes could be considered as safe for the selected cell lines.

**Figure 6:**
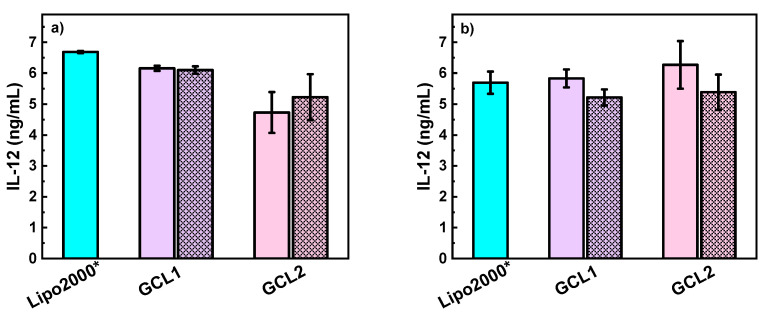
Transfection efficiency levels of therapeutic gene expressed as IL-12 (ng/mL) in COS-7 (**a**) and HeLa (**b**) cell lines in the presence of GCL/DOPE-pCMV-IL12 lipoplexes formed at ρ_eff_ = 5 (unstriped bars) and 10 (striped bars). The lipoplexes were incubated with cells for 48 h. The blue bar corresponds to commercial Lipo2000*, used here as positive control. The data represent the mean of three wells, and errors are within ±5%.

The transfection efficiency of the GCL/DOPE lipid mixtures was initially evaluated with the reporter gene pCMV-Luc. Supplementary Materials Figure S5 presents luciferase expression in COS-7 cells expressed in terms of ng luciferase/mg protein for the GCL/DOPE-pCMV-Luc lipoplexes at ρ_eff_ = 5 and 10. Clear differences in transfection efficiency were observed between the lipoplexes, as highlighted by this figure: (i) the GCL3 lipoplex shows low transfection levels at all formulations (ρ_eff_), and it was therefore discarded for the following study with the IL-12 therapeutic gene; (ii) the other two lipoplexes show acceptable (GCL2) and remarkable (GCL1) levels of transfection, with the latter exceeding even the results obtained with the control; and (iii) there are no appreciable differences (within error) between the two formulations at ρ_eff_ = 5 and 10, although a subtle improvement is observed with ρ_eff_ = 10. These results may be attributed to the following: (i) the high DOPE content in the GCL1/DOPE-pCMV-Luc and GCL2/DOPE-pCMV-Luc lipoplexes (both with α = 0.2), which has already demonstrated to improve the fusogenic properties of this type of nanovectors [37,64,65]; (ii) the positive effect on the transfection efficiency of the imidazole head groups in GCL1 and GCL2 (with a delocalized positive charge) compared to the quaternary ammonium groups in GCL3, as already found for these lipid-based nanovectors when transfecting pEGFP-C3 [37,38]; and (iii) the oxyethylene spacer, which is believed to be more cell friendly than the methylene spacer [37,39]. In fact, this evidence could be responsible for the improved transfection efficiency of GCL1/DOPE-pCMV-Luc compared to GCL2/DOPE-pCMV-Luc, although it seems that the nature of the polar head has a greater effect, in view of the poor performance shown by the GCL3 lipoplex with also an oxyethylene spacer. In any case, both lipoplexes were considered efficient enough to be further evaluated for the ultimate aim of this work: the transfection of therapeutic gene pCMV-IL12, as evaluated in COS-7 and HeLa cells by means of ELISA experiments. Figure 6a (COS-7) and 6b (HeLa) show the cytokine expression levels (ng IL-12/mL), revealing that both lipoplexes are capable of transfecting pCMV-IL12 with remarkable efficiency, comparable to or even surpassing that of commercial control Lipo2000* for some of the prepared formulations (ρ_eff_). Notice that the GCL1/DOPE-pCMV-IL12 lipoplex presents slightly better performance in COS7 cells, while GCL2/DOPE-pCMV-IL12 seems to work slightly better in HeLa cancer cells (mostly at ρ_eff_ = 5), a result of great interest considering the antitumor activity of IL-12. In any case, both results suggest that no compelling reasons exist to choose one of the lipoplexes over the other, since both demonstrate their ability to transfect the plasmid that encodes for the cytokine of interest, IL-12, once their formulations (ρ_eff_) have been optimized. It is worth noting that other GCL-type vectors, such as C_3_(C_16_His)_2_ or one based on trehalose, have also been found to successfully transfect pCMV-IL12 [53,66]. The reported results, together with those presented in this work, reinforce the potential of GCL-based vectors (assisted by a helper zwitterionic lipid like DOPE) as non-viral nanocarriers of therapeutic gene pCMV-IL12. Moreover, as concluded in the protein corona study, the corresponding GCL/DOPE-pCMV-IL12 lipoplexes have a clear tendency to adsorb proteins that improve its traffic in the bloodstream, such as lipoproteins and serum albumin. For all the above reasons, GCL-based nanocarriers in general, and those reported herein in particular, can be considered serious candidates for pIL-12 delivery and consequently in the design of further strategies for future medical applications.

## 4. Conclusions

Inappropriate activation of inflammatory processes can lead to many pathologies and diseases. Alzheimer’s disease, cancer or autoimmune diseases are examples related to chronic inflammation, while infectious diseases, like the one currently desolating the world (COVID-19), seem to be potentiated by an uncontrolled immune response. IL-12 has interesting immunomodulatory functions, acting as a bridge between innate response and adaptive immunity. Still, different approaches to deliver IL-12 remain an active area of research. Nanovectors that successfully and safely transport pIL-12 into cells are needed in clinical trials based on IL-12 gene therapies. With this in mind, we selected GCLs offering optimal results in previous studies and combined them with zwitterionic lipid DOPE to transport and deliver pCMV-IL12 into cells. The physicochemical study demonstrated the formation of positively charged lipoplexes with a reporter gene, pCMV-Luc. Furthermore, the ability of lipid mixtures to protect and compact pDNA was demonstrated by agarose gel electrophoresis, while cryo-TEM experiments revealed that the pDNA was complexed in a L_α_ multilamellar liotropic liquid crystal phase. The characterization of the GCL/DOPE-pDNA lipoplexes was completed with an extensive study on the profile of proteins surrounding the vector in the presence of human serum. The most abundant proteins, lipoproteins and serum albumin, seem to suggest an adequate behavior of these lipoplexes in blood fluids for future therapies. The three GCL/DOPE-pDNA lipoplexes demonstrated high biocompatibility, as shown by cytotoxicity studies on HeLa and COS-7. However, upon evaluation of the efficiency of transfection of the three lipoplexes with pCMV-Luc, only those containing imidazole groups in the polar heads (GCL1/DOPE-pCMV-Luc and GCL2/DOPE-pCMV-Luc) in combination with DOPE at α = 0.2 were found to be adequate. This feature reinforces the fusogenic properties of zwitterionic lipid DOPE and the capacity of imidazole groups to favor the transfection process. Finally, ELISA assays demonstrated the ability of GCL1/DOPE and GCL2/DOPE lipid mixtures to transfect the pCMV-IL12 therapeutic gene, with results comparable to those obtained with the Lipo2000* control. In particular, the results with GCL2/DOPE-pCMV-IL12 were not only similar to those of Lipo2000* but superior in the case of the HeLa cell tumor line. This finding is quite interesting since the PC of these lipoplexes showed a remarkable serum albumin fraction. This protein, in addition to inhibiting macrophage recognition, may also contribute to the accumulation of lipoplexes in tumors, where IL-12 may exert its antitumor activity. Definitely, these results encourage further and more specific studies to gain deeper insight into cytokine vectorization for advanced therapies and for the development of preventive vaccines, which will have a positive impact on the health of human beings worldwide. Exogenous pIL-12 has already shown positive effects as therapeutic agents, on its own or as an adjuvant in combination approaches. This study highlights the ability of certain non-viral lipid-type vectors (particularly GCLs) to transport plasmids encoding for IL-12 in different cell lines, which may further contribute to the design of new strategies to treat different diseases.

## Figures and Tables

**Table 1:** Size (D_h_) and polydispersity index (PDI) values for the GCL/DOPE-pCMV-Luc lipoplexes at ρ_eff_ = 5 and 10.

Lipoplexes	ρ_eff_ = 5		ρ_eff_ = 10	
	D_h_ (nm)	PDI	D_h_ (nm)	PDI
GCL1/DOPE-pCMV-Luc	265	0.39	181	0.36
GCL2/DOPE-pCMV-Luc	205	0.30	152	0.24
GCL3/DOPE-pCMV-Luc	202	0.36	185	0.39

Errors are less than 10%.

**Table 2:** Relative percentage of the top 25 most-abundant proteins in the PC surrounding the GCL/DOPE-pCMV-Luc lipoplex surfaces at ρ_eff_ = 5. On the right, the color scale from 0 (gray) to 11% abundance (red).

Proteins	%			
	GCL1	GCL2	GCL3	
Alpha-1-antitrypsin SV = 1				11
Alpha-1-antitrypsin SV = 3				
APOC4-APOC2				
Apolipoprotein A-I				
Apolipoprotein A-II				
Apolipoprotein A-IV				9
Apolipoprotein B-100				
Apolipoprotein C-I				
Apolipoprotein C-III				
Apolipoprotein D				
Apolipoprotein E				7
Apolipoprotein M				
C4b-binding protein alpha chain				
Complement C3				
Haptoglobin				
Haptoglobin-related protein				5
Hyaluronan-binding protein 2				
Ig heavy constant gamma 1 (Fragment)				
Ig kappa constant				
Ig lambda constant 3				
Inter-alpha-trypsin inhibitor heavy chain H1				3
Isoform 2 of Ig heavy constant mu				
Protein AMBP				
Prothrombin				
SAA2-SAA4 readthrough				
Serum albumin				1
Serum amyloid A-1 protein				
Trypsin				
Vitronectin				0

## Supplementary Materials

The following information is available online at https://www.mdpi.com/article/10.3390/pharmaceutics13050729/s1. Table S1: Effective charges of GCLs and pDNA and the values of electroneutrality ratio (m_L_/m_pDNA_)_ϕ_ for the lipoplexes studied in this work. Figure S1: Cryo-TEM micrographs of GCL2/DOPE-pCMV-Luc lipoplexes at ρ_eff_ = 5 and ρ = 0.2. Table S2: Relative percentage of proteins surrounding the surfaces of GCL/DOPE-pCMV-Luc lipoplexes, classified by their physiological function, by their molecular weight (MW) in kDa and by their isoelectrical point (pI) related with Figure 4. Figure S2: Classification of the most abundant proteins ((**a**) lipoproteins, (**b**) acute-phase, (**c**) others and (**d**) immunoglobulins proteins) found in the protein corona surrounding the surfaces of GCL/DOPE-pCMV-Luc lipoplexes. Figure S3: Classification of the proteins that constitute a minor fraction ((**a**) tissue leakage, (**b**) coagulation, and (**c**) complement proteins) present in the protein corona surrounding the surfaces of GCL/DOPE-pCMV-Luc lipoplexes. Figure S4: Relative percentage of the TOP 25 proteins found within the protein corona (PC) of GCL/DOPE-pCMV-Luc lipoplexes formed at ρ_eff_ = 5, classified by their molecular weight, MW in kDa (**a**), by their isoelectric point, pI (**b**) and by their physiological function (**c**). Figure S5: Transfection efficiency levels expressed as ng luciferase/mg protein in COS-7 cell line in presence of GCL/DOPE-pCMV-Luc lipoplexes formed at ρ_eff_ = 5 (unstriped bars) and 10 (striped bars).
