# Supplementary material for: Gemini Cationic Lipid-Type Nanovectors Suitable for the Transfection of Therapeutic Plasmid DNA Encoding for Pro-Inflammatory Cytokine Interleukin-12
Source: Pharmaceutics. 2021 May 15;13(5):729. doi: 10.3390/pharmaceutics13050729 (PMC8156092; doi:10.3390/pharmaceutics13050729)
Supplement: Supplementary file 1 [file pharmaceutics-13-00729-s001.zip › pharmaceutics-1197894-supplementary.pdf]

pharmaceutics
MDPI

Supplementary Materials: Gemini Cationic Lipid-Type Nanovectors Suitable for the Transfection of Therapeutic Plasmid DNA Encoding for Pro-Inflammatory Cytokine Interleukin-12

Natalia Sánchez-Arribas, María Martínez-Negro, Clara Aicart-Ramos, Conchita Tros de Ilarduya, Emilio Aicart, Andrés Guerrero-Martínez and Elena Junquera

1. Physicochemical Characterization

Table S1. Effective charges of GCLs and pDNA and the values of electroneutrality ratio $(m_L/m_{pDNA})_\phi$ for the lipoplexes studied in this work.

System	GCL1/DOPE-pDNA	GCL2/DOPE-pDNA	GCL3/DOPE-pDNA
$q^{+}_{eff,GCL}$	1.9 [a]	1.7 [b]	1.8 [c]
$q^{-}_{eff,pDNA}/bp$	−1.5	−1.3	−1.4
$(m_L/m_{pDNA})_\phi$	4.5	4.5	1.6

Values estimated with a 5% error. The $q^{+}_{eff,GCL}$ values were obtained in previous studies [1–3], considering $q^{-}_{nom,ctDNA} = -2.0$ / bp. The $q^{-}_{eff,pDNA}/bp$ values were obtained from plot of ζ-potential as a function of m_L/m_{pDNA} (Figure 1) and working with these $q^{+}_{eff,GCL}$ values. [a] Ref. [1], [b] Ref. [2], and [c] Ref. [3].

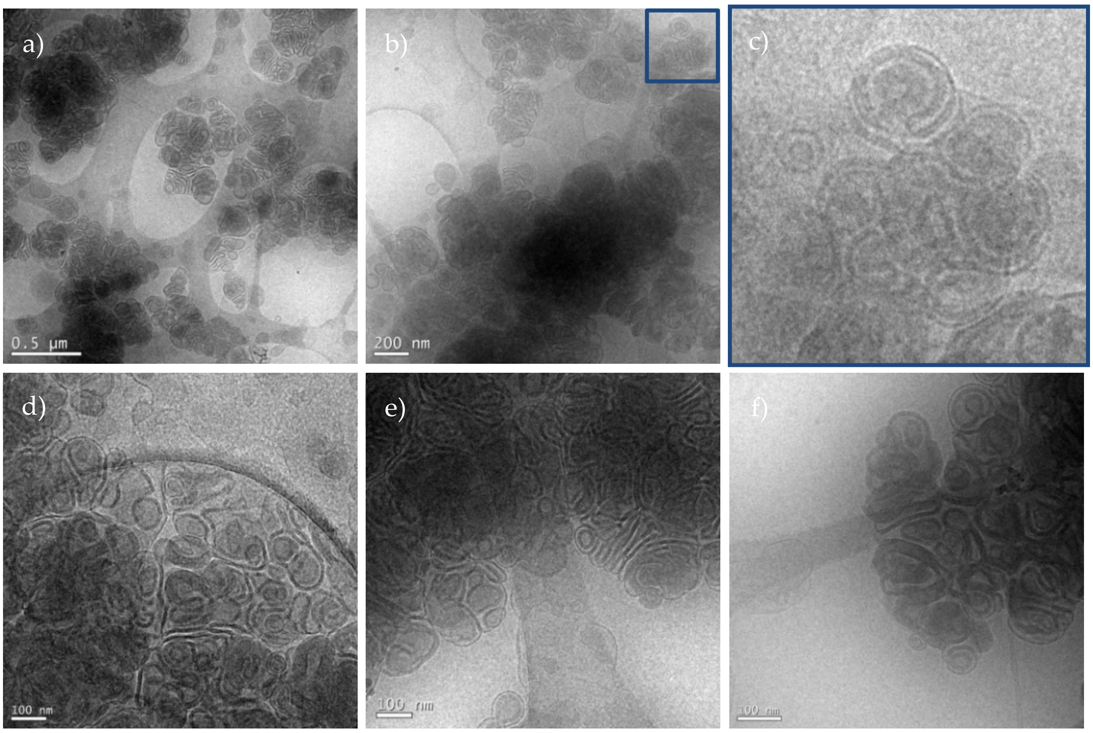

Figure S1. Cryo-TEM micrographs of GCL2/DOPE-pCMV-Luc lipoplexes at $\rho_{eff} = 5$ and $\alpha = 0.2$. Scale bars of micrographs are 500 (**a**), 200 (**b**) and 100 (**d**–**f**) nm. Panel (**c**) shows a zoom view of the blue square in panel (**b**).

2. Protein Corona Characterization

Table S2. Relative percentage of proteins surrounding the surfaces of GCL/DOPE-pCMV-Luc lipoplexes, classified by their physiological function, by their molecular weight (MW) in kDa and by their isoelectric point (pI) related with Figure 4.

**MW**	%		
	GCL1	GCL2	GCL3
>300	5.7	2.7	7.7
150–300	3.6	2.6	3.2
100–150	4.4	1.7	2.7
80–100	1.3	0.5	1.0
70–80	3.2	2.6	2.7
60–70	10.1	13.5	9.1
50–60	7.1	6.2	7.6
40–50	11.3	10.6	14.5
30–40	12.3	17.0	14.8
20–30	17.0	17.4	14.7
<20	24.1	25.1	22.0

**pI**	%		
	GCL1	GCL2	GCL3
>9	0.9	0.7	0.7
08_09	16.8	18.3	14.6
07_08	21.4	16.0	22.1
06_07	32.8	39.7	34.2
05_06	27.8	24.9	28.2
<5	0.3	0.2	0.1

**Physiological Function**	%		
	GCL1	GCL2	GCL3
Lipoproteins	38.5	37.3	37.9
Acute-phase	19.6	15.0	19.8
Immuno-globulins	12.4	14.2	16.5
Tissue Leak-age	2.9	3.2	3.1
Coagulation	6.9	4.7	6.0
Complement	8.0	5.4	4.0
Others	11.7	20.0	12.8

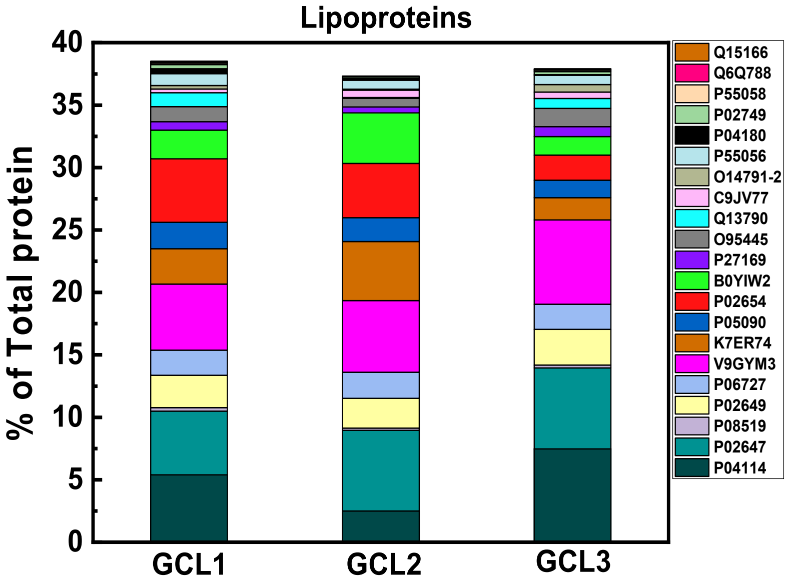
Lipoproteins
% of Total protein
0
5
10
15
20
25
30
35
40
GCL1
GCL2
GCL3
Q15166
Q6Q788
P55058
P02749
P04180
P55056
O14791-2
C9JV77
Q13790
O95445
P27169
B0YIW2
P02654
P05090
K7ER74
V9GYM3
P06727
P02649
P08519
P02647
P04114

(a)

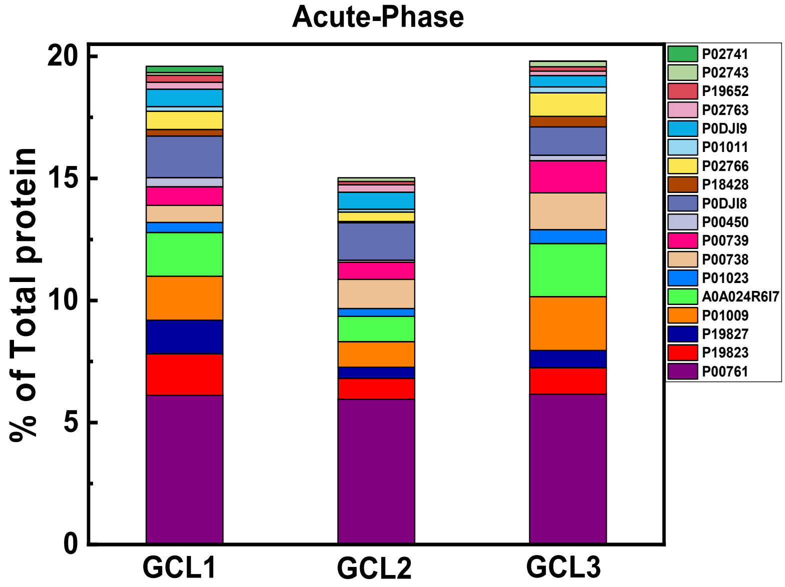
Acute-Phase
% of Total protein
0
5
10
15
20
GCL1
GCL2
GCL3
P02741
P02743
P19652
P02763
P0DJI9
P01011
P02766
P18428
P0DJI8
P00450
P00739
P00738
P01023
A0A024R6I7
P01009
P19827
P19823
P00761

(b)

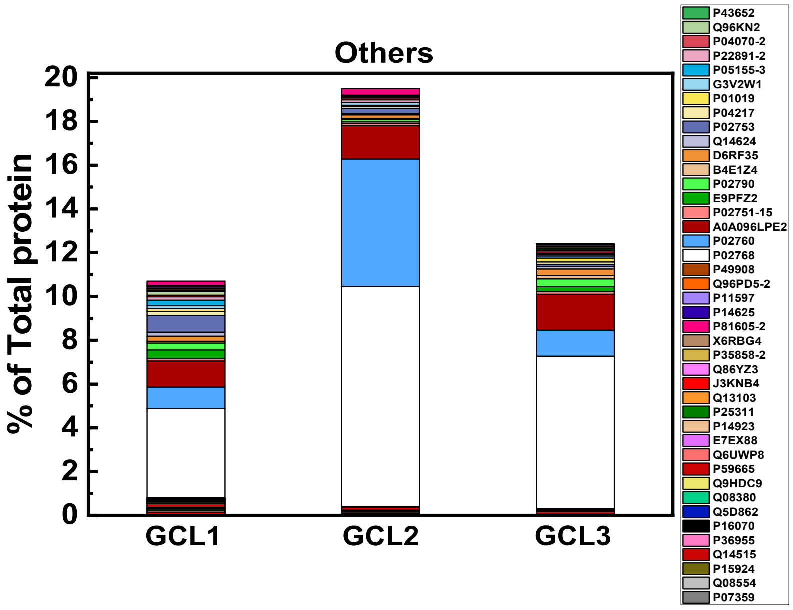

(**c**)

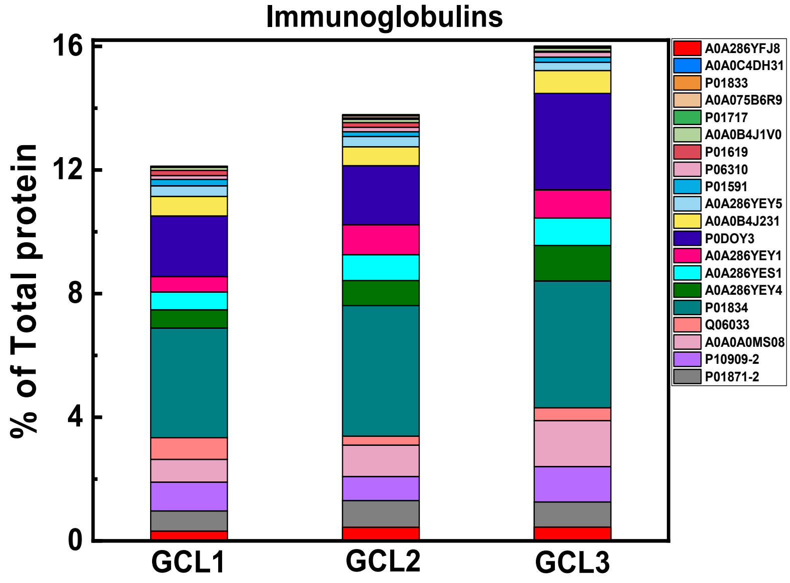

(**d**)

Figure S2. Classification of the most abundant proteins ((**a**) lipoproteins, (**b**) acute-phase, (**c**) others and (**d**) immunoglobulins proteins) found in the protein corona surrounding the surfaces of GCL/DOPE-pCMV-Luc lipoplexes.

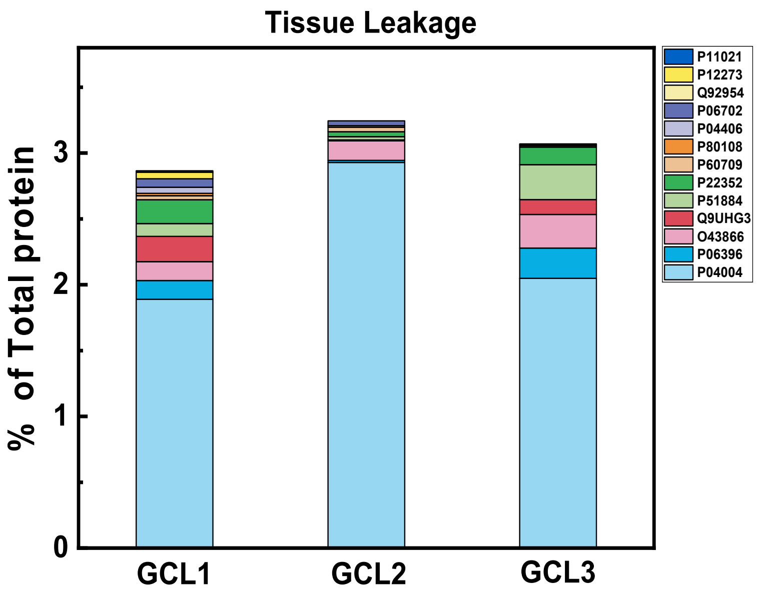

Tissue Leakage
% of Total protein
0
1
2
3
GCL1
GCL2
GCL3
P11021
P12273
Q92954
P06702
P04406
P80108
P60709
P22352
P51884
Q9UHG3
O43866
P06396
P04004

(a)

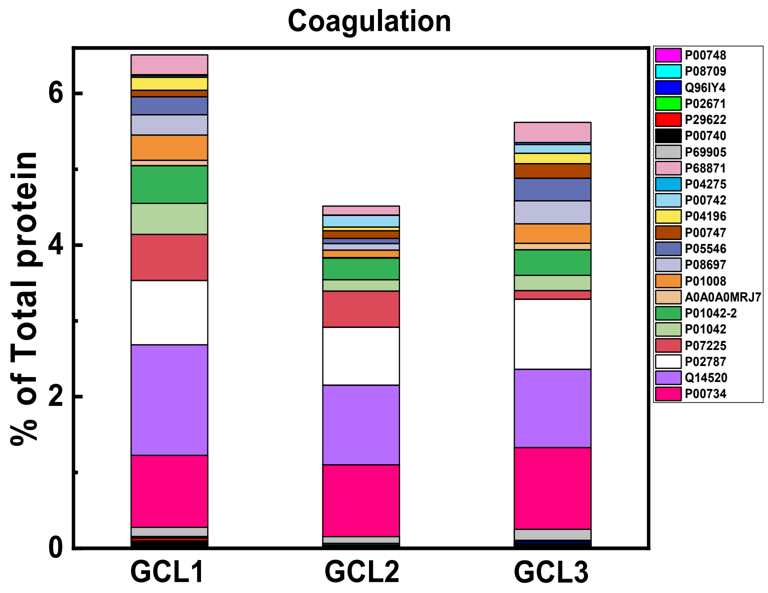

Coagulation
% of Total protein
0
2
4
6
GCL1
GCL2
GCL3
P00748
P08709
Q96IY4
P02671
P29622
P00740
P69905
P68871
P04275
P00742
P04196
P00747
P05546
P08697
P01008
A0A0A0MRJ7
P01042-2
P01042
P07225
P02787
Q14520
P00734

(b)

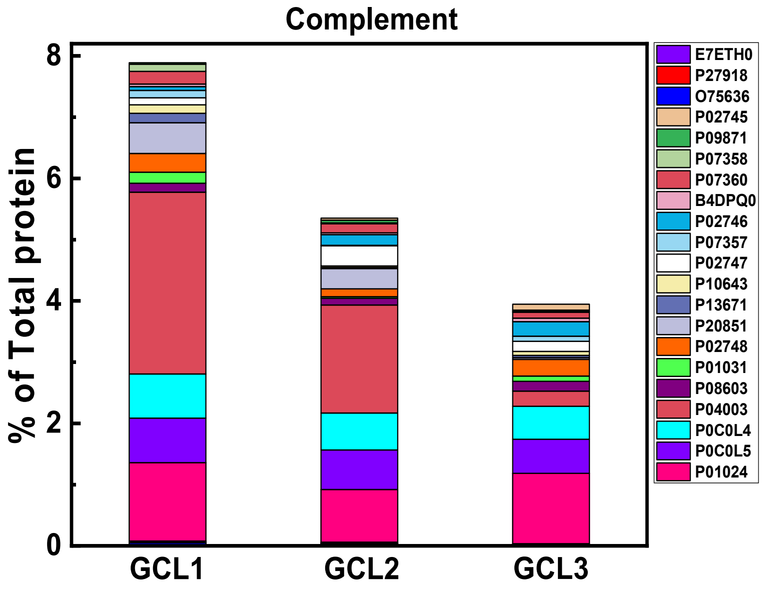

(**c**)

Figure S3. Classification of the proteins that constitute a minor fraction ((**a**) tissue leakage, (**b**) coagulation, and (**c**) complement proteins) present in the protein corona surrounding the surfaces of GCL/DOPE-pCMV-Luc lipoplexes.

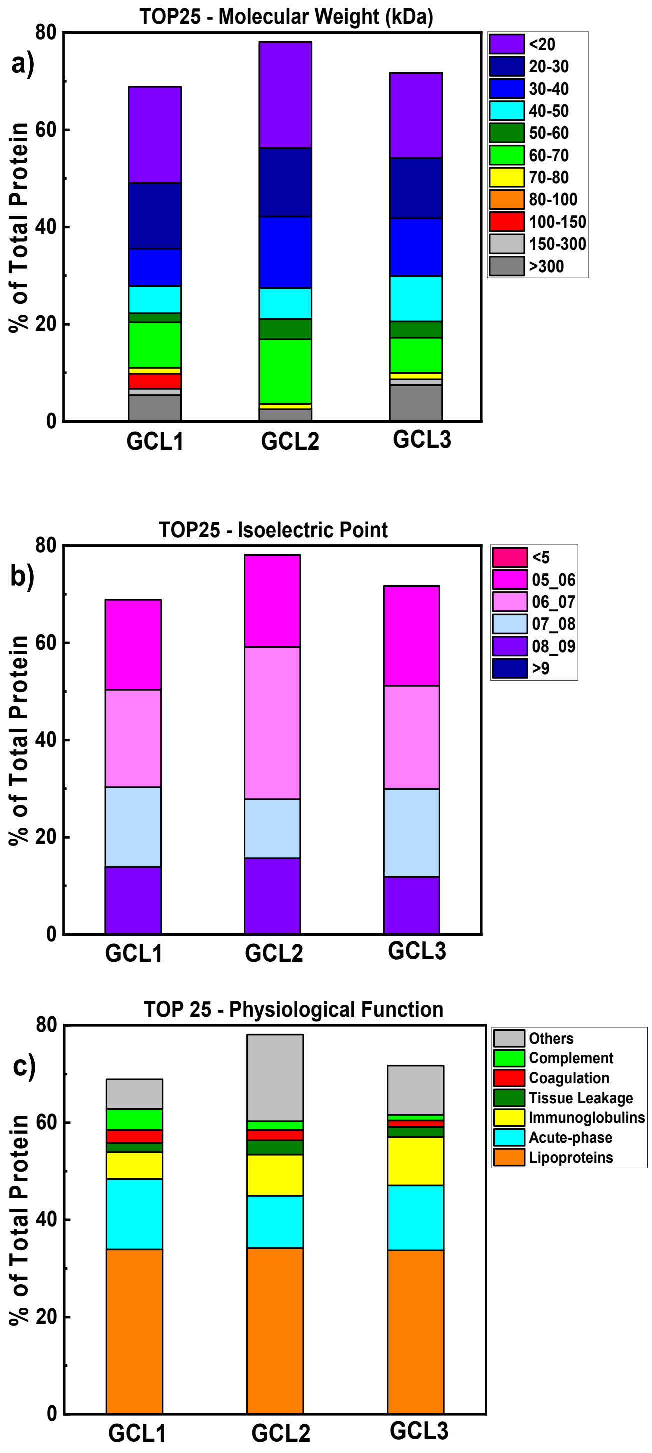

Figure S4. Relative percentage of the TOP 25 proteins found within the protein corona (PC) of GCL/DOPE-pCMV-Luc lipoplexes formed at ρ_{eff} = 5, classified by their molecular weight, MW in kDa (**a**), by their isoelectric point, pI (**b**) and by their physiological function (**c**).

3. *In Vitro* Studies

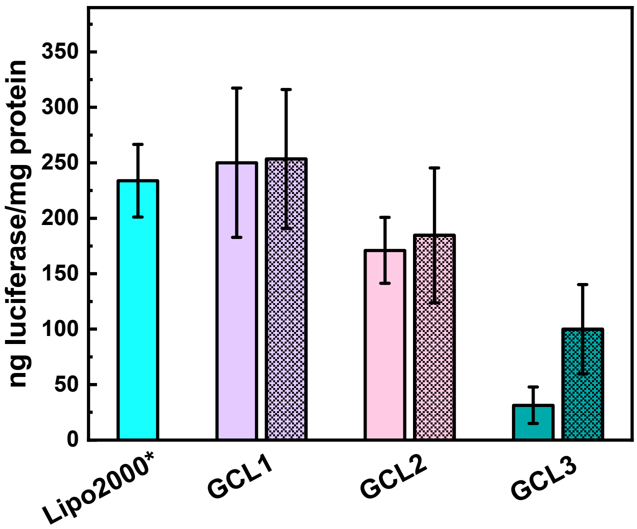

Figure S5. Transfection efficiency levels expressed as ng luciferase/mg protein in COS-7 cell line in presence of GCL/DOPE-pCMV-Luc lipoplexes formed at ρ_{eff} = 5 (unstriped bars) and 10 (striped bars). Lipoplexes were incubated with cells during 48 h. Blue bar corresponds to commercial control Lipo2000*, used as positive control. Data represent the mean of three wells and errors are within ± 5 %.

References

1. Kumar, K.; Barrán-Berdón, A.L.; Datta, S.; Muñoz-Úbeda, M.; Aicart-Ramos, C.; Kondaiah, P.; Junquera, E.; Bhattacharya, S.; Aicart, E. A delocalizable cationic headgroup together with an oligo-oxyethylene spacer in gemini cationic lipids improves their biological activity as vectors of plasmid DNA. *J. Mat. Chem. B* **2015**, *3*, 1495–1506, doi:10.1039/c4tb01948b.
2. Misra, S.K.; Muñoz-Úbeda, M.; Datta, S.; Barrán-Berdón, A.L.; Aicart-Ramos, C.; Castro-Hartmann, P.; Kondaiah, P.; Junquera, E.; Bhattacharya, S.; Aicart, E. Effects of a delocalizable cation on the headgroup of gemini lipids on the lipoplex-type nano-aggregates directly formed from plasmid DNA. *Biomacromolecules* **2013**, *14*, 3951–3963, doi:10.1021/bm401079h.
3. Barrán-Berdón, A.L.; Misra, S.K.; Datta, S.; Muñoz-Úbeda, M.; Kondaiah, P.; Junquera, E.; Bhattacharya, S.; Aicart, E. Cationic gemini lipids containing polyoxyethylene spacers as improved transfecting agents of plasmid DNA in cancer cells. *J. Mat. Chem. B* **2014**, *2*, 4640–4652, doi:10.1039/C4TB00389F.
